# The Work of the Holy Ghost

**Published:** 1887-07-16

**Authors:** 


### WORDS OF CONSOLATION.

[Communications for this column should be addressed, " Chaplain," care of the Editor of THE HOSPITAL, who will gratefully welcome any suggestions or inquiries from matrons, nurses, and the staff generally.]

### XLI.—THE WORK OF THE HOLY GHOST.

ANOTHER branch of the Holy Spirit's work is signified by the title of the Comforter. The comfort, like the guidance, belongs especially to those whom He has already made steadfast disciples of Christ. He is the Comforter of the faithful, the Comforter of those who have yielded to His leadership. Herein do Christians discover the value of their religion, for just when they most need the peace which the world cannot give, Christ is true to His promise, and returns unto them and makes Himself known in the presence of the Comforter. Let us be sure of this; the consolations of religion are not to be expected apart from its practice. "If ye love me keep my commandments; and I will pray the Father, and he shall give you another Comforter that he may abide with you for ever; even the Spirit of truth whom the world cannot receive" (John xiv, 15, 16). Mark the conditions upon which the Comforter is manifested—obedience to the commandments, for love of Jesus. The worldly-minded, who have never given the Holy Spirit any welcome as Convincer, Teacher, or Guide, cannot either receive Him as the Comforter. This is taught us as plainly by experience as by the Bible. We see disobedient, careless, unspiritual people visited with sickness or sorrow, deprived at the same time of the consolations which their worldly or social position once could give them, and it is all too plain that they have no idea of receiving comfort from any other source. How miserable, how full of complaints, how morose they become, because the Holy Ghost is not present to cheer them in the darkness, or if present in the stern rebukes of conscience, for that very reason He cannot at the same time arrest the bitter fretfulness of the worldly mind—"Whom the world *cannot* receive." God's ministers can tell, and hospital chaplains, how often they have to deplore their own complete failure as comforters, because it has been all too plain that *the* Comforter was not present, as such, in some heart they have much wanted to cheer. It is natural, and perhaps right, that the sick should always have what are commonly called the " consolations" of religion offered them, at least to begin with; for we are not called upon to constitute ourselves judges of who are capable of receiving them, and who are not. But we must remember that in a very large number of cases there are deep impressions which the Holy Ghost is lovingly bent on making on the hard surface of the heart before He can proceed to the ministry of consolation. We cannot "prevent" Him in this ministry. Not until He has been received, welcomed, obeyed, can we expect our consolations to be much appreciated.

(*To be continued.*)

